# Classification of Multiple DNA Dyes Based on Inhibition Effects on Real-Time Loop-Mediated Isothermal Amplification (LAMP): Prospect for Point of Care Setting

**DOI:** 10.3389/fmicb.2019.02234

**Published:** 2019-10-15

**Authors:** Than Linh Quyen, Tien Anh Ngo, Dang Duong Bang, Mogens Madsen, Anders Wolff

**Affiliations:** ^1^Department of Biotechnology and Biomedicine, Technical University of Denmark (DTU-Bioengineering), Lyngby, Denmark; ^2^Laboratory of Applied Micro and Nanotechnology (LAMINATE), National Food Institute, Technical University of Denmark (DTU-Food), Lyngby, Denmark

**Keywords:** loop-mediated isothermal amplification (LAMP), real-time LAMP, classification, *Salmonella*, inhibitory effects, DNA dye

## Abstract

LAMP has received great interest and is widely utilized in life sciences for nucleic acid analysis. To monitor a real-time LAMP assay, a fluorescence DNA dye is an indispensable component and therefore the selection of a suitable dye for real-time LAMP is a need. To aid this selection, we investigated the inhibition effects of twenty-three DNA dyes on real-time LAMP. Threshold time (T_t_) values of each real-time LAMP were determined and used as an indicator of the inhibition effect. Based on the inhibition effects, the dyes were classified into four groups: (1) non-inhibition effect, (2) medium inhibition effect, (3) high inhibition effect, and (4) very high inhibition effect. The signal to noise ratio (SNR) and the limit of detection (LOD) of the dyes in groups 1, 2, and 3 were further investigated, and possible inhibition mechanisms of the DNA dyes on the real-time LAMP are suggested and discussed. Furthermore, a comparison of SYTO 9 in different LAMP reactions and different systems is presented. Of the 23 dyes tested, SYTO 9, SYTO 82, SYTO 16, SYTO 13, and Miami Yellow were the best dyes with no inhibitory effect, low LOD and high SNR in the real-time LAMP reactions. The present classification of the dyes will simplify the selection of fluorescence dye for real-time LAMP assays in point of care setting.

## Introduction

Over the last 18 years, LAMP has been used widely in the laboratory and in point of care (POC) settings to analyze nucleic acid as well as to detect pathogens (Notomi et al., 2000; Njiru, 2012; Seki et al., 2018). LAMP is faster than PCR (Espy et al., 2006; de Paz et al., 2014) and can be performed under simpler conditions, i.e., at a constant temperature in a range of 60–65°C without the thermal cycling required for PCR (Notomi et al., 2000; Sun et al., 2015). LAMP has advantages such as being rapid, sensitive, specific, simple to perform, less expensive (Njiru, 2012; Velders et al., 2018), and less affected by inhibitors (Stedtfeld et al., 2014; Kosti et al., 2015). LAMP has therefore been considered an attractive method for POC systems (de Paz et al., 2014; Sun et al., 2015). The LAMP reaction produces large amounts of amplified product (dsDNA) and the by-product (magnesium pyrophosphate) that allow visualization of results using a real-time PCR machine (Aoi et al., 2006; Chen and Ge, 2010) or a turbidimeter (Mori et al., 2004), and can even be detected by the naked eye when using appropriate DNA staining techniques (Amin Almasi, 2012; Xie et al., 2014).

Real-time LAMP has been employed since it is not only effective for DNA amplification under isothermal conditions but also simultaneously offers detection, which can be quantitative for e.g., the monitoring of specific gene expression (Mori et al., 2004; Cai et al., 2008; Chen and Ge, 2010; Tourlousse et al., 2012; Cao et al., 2015) or for detection of bacterial concentrations (Nixon et al., 2014). Measurement of turbidity has been used in real-time LAMP (Takano et al., 2019) and a commercial detection device is available (Eiken Chemical, Co., Ltd., Japan). The real-time LAMP turbidity is based on quantification of an increase of magnesium pyrophosphate precipitate as a by-product of the LAMP reaction (Mori et al., 2004). However, the turbidity-based detection method is 10 times less sensitive than that of fluorescence detection (Chen and Ge, 2010). Molecular beacon probes in the F1-B1 region and FRET-like fluorogenic probes in the loop region have been designed and tested, but the amplification efficiency of the assays was reported to be significantly reduced (Cai et al., 2008). The use of DNA dyes is therefore considered an excellent alternative detection method in real-time LAMP since it is simple and it can also enhance the sensitivity of the assay as compared to the turbidity measurement (Cao et al., 2015). In addition, it has been shown that DNA dyes such as SYTO 82 could be used for real-time LAMP using a low-cost charge-coupled device (CCD) based fluorescence imaging system that could be comparable to a commercial real-time PCR instrument (Ahmad et al., 2011). Sun et al. (2015) also reported the use of SYTO 62 for real-time LAMP in an integrated LOC system. Those reports showed the potential of using DNA dyes in POC devices.

Several DNA dyes such as SYBR Green I (Patel et al., 2013), SYTO 9 (Chen and Ge, 2010; Patel et al., 2013), SYTO 82 (Ahmad et al., 2011), YOPRO-1 (Hara-Kudo et al., 2005) and Pico Green (Tian et al., 2012) have been utilized in real-time LAMP. However, none of these studies reported any further investigation on inhibitory effects of the dyes on the real-time LAMP assay. We previously investigated the inhibitory effects of six dyes (SYTO 24, SYTO 62, SYTO 82, SYBR Green I, Eva Green and SYBR Safe) at 5 μM concentration on the real-time LAMP assay (Sun et al., 2015). Seyrig et al. (2015) and Oscorbin et al. (2016) demonstrated the inhibitory effects of fourteen dyes belonging to green dyes (SYBR Green I, Pico Green, Eva Green, Calcein, SYTO 9, SYTO 13, and SYBR Gold) and orange dyes (SYTO 80, SYTO 81, SYTO 82, SYTO 83, SYTO 84, SYTO 85, and SYTOX Orange) on real-time LAMP (Seyrig et al., 2015; Oscorbin et al., 2016). However, these studies were performed on a narrow range of two groups of DNA dyes. Moreover, previous publications on the use of different dyes in real-time LAMP have not included detailed data of fluorescence signal strength or investigation of LAMP reaction efficiencies – a critical aspect of a real-time reaction. Furthermore, it is difficult to predict the behavior of different dyes in real-time LAMP due to the lack of detailed structural information of most of the dyes.

In this study, we thoroughly investigated the inhibitory effects and classified 23 DNA dyes that span a wide range of four groups of dyes with different optical properties in a real-time LAMP assay for detection of *Salmonella* Enteritidis. The dyes were used at various concentrations. All the dyes were further evaluated for their fluorescence intensity at the end of the LAMP assay at different dye concentrations. Also, the detection limit of the real-time LAMP assay was investigated and compared when using different dyes from different groups. Furthermore, the inhibition effect of SYTO 9 using different LAMP reactions and different systems has been studied. The classification of the dyes into four groups based on inhibition effect does not only suggest which dyes have the greatest potential for the development of real-time LAMP that can be integrated into a POC device, but also provides essential information for selecting DNA dyes for other applications in real-time LAMP detection.

## Materials and Methods

### DNA Preparation

*Salmonella* Enteritidis (*S.* Enteritidis) strain CCUG-32352 (originating from the University of Gothenburg Culture Collection) was obtained from the culture collection of National Food Institute, Technical University of Denmark (DTU-Food). *S.* Enteritidis genomic DNA was isolated using DNeasy Blood and Tissue kit (Qiagen, Germany) according to the supplier instructions. The DNA concentration was determined by Nanodrop 1000 (Thermo Scientific, United States) and the DNA preparation was stored at minus 20°C before use.

### Primers and Real-Time LAMP Conditions

We designed a *Salmonella* LAMP primer set (Supplementary Table S1) based on the hilA gene sequence alignment of *S. enteritica* (NCBI GenBank accession no. CP010.280.1) using the PrimerExplorer V4 (Eiken Chemical Co. Ltd., Tokyo, Japan). A *Campylobacter* primer set targeting the Cj0414 gene of *Campylobacter jejuni* was also selected for this study (Supplementary Table S1; Yamazaki et al., 2009).

The LAMP assay was carried out in 10 μl master mixture containing 0.2 μM of F3; 0.2 μM of B3; 1.4 μM of FIP; 1.4 μM of BIP; 0.8 μM of LF; 0.8 μM of LB; 1.4 mM dNTP mix (DNA Technology, Aarhus, Denmark), 0.5 M Betaine (Sigma-Aldrich, Denmark), 4 U of Bst 2.0 DNA polymerase (New England BioLabs), 1× isothermal amplification buffer (comprising 20 mM Tris–HCl, 10 mM (NH4)_2_SO4, 50 mM KCl, 2 mM MgSO4, and 0.1% Tween^®^ 20, pH 8.8), various concentrations ranging from 0.5 μM to 10 μM of each dye that included SYTO 9, SYTO 13, SYTO 16, SYTO 24, SYTO 60, SYTO 62, SYTO 64, SYTO 82, SYBR Green I, SYBR Gold, YOPRO1, TOTO1, TOTO3, BOBO3, POPO3, and TOPRO3 (Invitrogen, United States); Eva Green (Biotium, United States); Boxto (TATA Biocentre, Sweden); Miami Green, Miami Yellow, and Miami Orange (Kerafast, United States), Pico 488 (Lumiprobe, Germany) and Nuclear Green DCS1 (Abcam, United Kingdom), sterilized water and DNA template.

All the LAMP assays were conducted in a DNA engine thermocycler with a Chromo4 real-time detector (Bio-Rad Laboratories Inc., Hercules, CA, United States) using thin-walled 100 μl white PCR tube strips (Abgene, Surrey, United ingdom), in an Mx3005P (Stratagene, AH diagnostics, Denmark), and in a Piko real-time PCR system (Thermo Fisher Scientific, Finland). The reactions were performed at 65°C for 60 min and the reactions were then terminated by heating to 90°C for 10 min. The fluorescent signal was recorded every minute of amplification. Excitation and emission wavelengths of all the dyes and the fluorescence recording channels used for the dyes in this study are listed in Supplementary Table S2. The excitation and emission of each channel are shown in Supplementary Table S3.

### Gel Electrophoretic Analysis

After completion of real-time LAMP reactions, the LAMP amplified products were confirmed by gel electrophoresis. Five μl of each LAMP amplified products were loaded on 2% agarose gel containing 1× of SYBR^®^ Safe DNA Gel Stain (Invitrogen, Life Technologies, United States). The gel electrophoresis was carried out at 25 volts for 30 min and was visualized under UV light using a BioSpectrum^®^ AC imaging system (AH diagnostics, Denmark).

### Sensitivity Experiments

The sensitivity of real-time LAMP was performed at an optimal concentration for each of the dyes SYTO 9, SYTO 13, SYTO 16, SYTO 64, Boxto, Miami Yellow, TOPRO 3, SYTO 60, SYTO 62, Eva Green, SYBR Green I and Nuclear Green DCS1. Ten-fold dilutions of *S.* Enteritidis chromosomal DNA ranging from 2000 to 0.02 pg were prepared. One microliter of each DNA concentration was used as a template for LAMP reactions in the sensitivity test. It has been estimated that 2000 pg of *S.* Enteritidis genomic DNA is corresponding to 3.86 × 10^5^ copies of *S.* Enteritidis genome (Chin et al., 2016). The sensitivity experiments were repeated four times. The lowest DNA concentration that gave a positive result in three experiments was considered as the limit of detection (LOD) of the assay.

### Data Analysis

Similar to the threshold cycle (C_t_) in a real-time PCR (Bustin et al., 2009), the threshold time value (T_t_) has been used to determine the real-time LAMP efficiency (Kubota et al., 2011). The T_t_ was defined as the time required for the fluorescence to reach a threshold value (F_t_). F_t_ was determined using equation 1 (eq. 1), which was based on the starting fluorescence value (F_i_) plus the average fluorescence value ($\Delta \,\bar{\text{F}}$) and three times of the standard deviation of the fluorescence values (3s_ΔF_) observed in triplicate negative reactions without DNA template (Kubota et al., 2011).

(1) $$F_{t}\,\text{=}\,F_{i}+\Delta \,\bar{F}+3\,s_{\Delta \,F}$$

Signal-to-noise ratio was determined and used to analyze the fluorescence intensity of the dyes in real time. The SNR was calculated by the fluorescence signal (arbitrary unit) (X) minus the average baseline signal in the first five points (μ) and divided by the standard deviation of the baseline signal at the first five points (σ) as in equation 2 (eq. 2) (Seyrig et al., 2015). The fluorescence signal of a real-time LAMP reaction was analyzed at an optimal concentration of each dye.

(2) S⁢N⁢R⁢=⁢(X-μ)/σ

To evaluate the speed of the real-time LAMP reactions for rapid detection, we determined the doubling time (DT) for double-stranded DNA assuming exponential amplification (Keremane et al., 2015; Kubota and Jenkins, 2015). The DT was calculated by multiplying the negative log (2) with the slope of threshold time versus log DNA concentration using equation 3 (eq. 3).

(3) DT⁢=-log⁢(2)×slope

## Results

### Inhibition Effect of DNA Dyes on Real-Time LAMP

The inhibition effect of the dyes on real-time LAMP reaction was investigated using different concentrations of each dye in the real-time LAMP assay. The efficiency of a real-time LAMP was evaluated based on the T_t_ value, which was used as an indicator of the inhibitory effects because an increase of the inhibitory effects would raise the T_t_ value. To define the inhibitory effect, the T_t_ values were plotted against the dye concentrations. The slope of the plot based linear relationship was used for further indicating the degree of the inhibition. An optimal concentration for each dye was defined as the concentration that resulted in the shortest T_t_ value. In addition, besides the determination of the T_t_ values, agarose gel electrophoresis was performed to confirm the LAMP amplification in case the dye used did not give a fluorescence signal in the real-time LAMP reaction.

**FIGURE 1:**
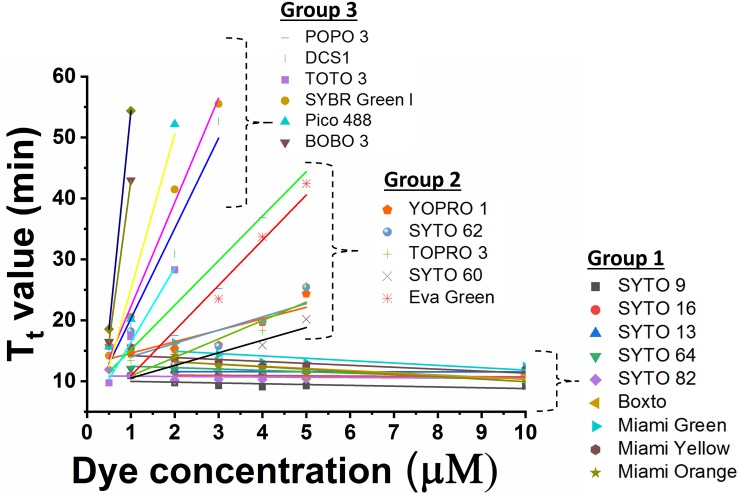
Comparison of T_t_ value against dye concentration for 20 dyes which exhibited fluorescence in real-time LAMP in presence of 2 ng DNA *S.* Enteritidis per reaction. The slope of the line indicates the degree of inhibition in real-time LAMP reaction.

In the real-time LAMP reactions using the 23 dyes, fluorescent signals were observed for 20 of these dyes including SYTO 9, SYTO 13, SYTO 16, SYTO 64, SYTO 82, Boxto, Miami Green, Miami Yellow, Miami Orange, YOPRO 1, SYTO 62, TOPRO 3, SYTO 60, EvaGreen, POPO 3, DCS1, SYBR Green I, BOBO 3, Pico 488, and TOTO 3. The T_t_ values of these 20 dyes were calculated and plotted against the dye concentrations at an initial DNA template concentration of 2 ng of DNA *S.* Enteritidis per reaction (Figure 1 and Supplementary Figures S1–S20). In contrast, no fluorescent signals were observed with the remaining 3 dyes (TOTO 1, SYTO 24, and SYBR Gold) in the real-time LAMP reaction (Supplementary Figures S21–S23). According to the results of the real-time LAMP reaction and slopes of linear relationship of all these dyes, the inhibitory effect of these dyes on the real-time LAMP reaction was classified into four different groups: (1) non-inhibition effect, (2) medium inhibition effect, (3) high inhibition effect, and (4) very high inhibition effect (Figure 1 and Table 1).

**TABLE 1:** Summary of the results obtained in this study and references.

**Dyes**	**Inhibitory effect**	**Optimal dye concentrati-on^1^**	**T_t_ value at optimal dye concentration (min)^1^**	**Maximum T_m_ shift**	**DNA binding affinity (References)**	**Binding selectivity (References)**
**Group 1: No inhibition**						
SYTO 9	No	2–10 μM	9.240.26	Low^2^ (Gudnason et al., 2007)	Low (Haugland, 2010)	dsDNA/ssDNA (Horáková et al., 2011)
SYTO 13	No	2–10 μM	11.010.01	Low^2^ (Gudnason et al., 2007)	Low (Haugland, 2010)	dsDNA/ssDNA (Horáková et al., 2011)
SYTO 16	No	2–10 μM	9.880.23	Low^2^ (Gudnason et al., 2007)	Low (Haugland, 2010)	dsDNA/ssDNA (Zhang et al., 2011)
SYTO 64	No	4–10 μM	9.620.21	Low^2^ (Gudnason et al., 2007)	Low (Haugland, 2010)	dsDNA/ssDNA (Erve et al., 2006)
SYTO 82	No	2–10 μM	10.200.29	Low^2^ (Gudnason et al., 2007)	Low (Haugland, 2010)	dsDNA/ssDNA (Erve et al., 2006)
Boxto	No	4–10 μM	11.040.22	Low^3^ Supplementary Figure S26	Lower than YOPRO (Eriksson, 2003)	dsDNA/very low affinity to ssDNA (Ahmad, 2007)
Miami Green	No	4–10 μM	13.670.30	Low^3^ Supplementary Figure S26	–	DNA targeting (Wilson et al., 2013)
Miami Yellow	No	2–10 μM	11.470.35	Low^3^ Supplementary Figure S26	–	DNA targeting (Wilson et al., 2013)
Miami Orange	No	4–10 μM	11.670.30	Low^3^ Supplementary Figure S26	–	DNA targeting (Pitter et al., 2015)
**Group 2: Medium inhibition**						
YOPRO 1	Medium	1 μM	14.840.38	Medium^2^ (Gudnason et al., 2007)	High (Haugland, 2010)	dsDNA/ssDNA (Haugland, 2010)
SYTO 62	Medium	2 μM	11.940.23	Medium^2^ (Gudnason et al., 2007)	Low (Haugland, 2010)	dsDNA/ssDNA (Erve et al., 2006)
TOPRO 3	Medium	2 μM	10.970.17	Medium^2^ (Gudnason et al., 2007)	High (Haugland, 2010),	dsDNA/ssDNA^31^
SYTO 60	Medium	2 μM	11.150.19	Medium^2^ (Gudnason et al., 2007)	Low (Haugland, 2010)	dsDNA/ssDNA (Erve et al., 2006)
Eva Green	Medium	1×	15.830.09	Low^3^ Supplementary Figure S26	Lower than SYBR Green I (Mao et al., 2007)	dsDNA > ssDNA (Mao et al., 2007) dsDNA/ssDNA (Horáková et al., 2011)
**Group 3: High inhibition**						
POPO3	High	0.5 μM	15.670.38	Low^2^ (Gudnason et al., 2007)	Very high (Haugland, 2010)	dsDNA/ssDNA (Haugland, 2010)
NG-DCS1	High	0.5 μM	9.270.18	High^3^ Supplementary Figure S26	High (AAT Bioquest, 2016)	DsDNA (AAT Bioquest, 2016)
SYBR Green I	High	0.5×	15.220.23	High^2^ (Gudnason et al., 2007; Radvanszky et al., 2015)	Very high (Haugland, 2010)	dsDNA > ssDNA (Haugland, 2010; Tuma et al., 1999)
BOBO3	High	0.5 μM	17.340.40	Low^2^ (Gudnason et al., 2007)	Very high (Haugland, 2010)	dsDNA/ssDNA (Haugland, 2010)
TOTO 3	High	0.5 μM	14.240.54	Low^2^ (Gudnason et al., 2007)	Very high (Haugland, 2010)	dsDNA/ssDNA (Haugland, 2010)
Pico 488	High	0.5 μM	16.490.41	Medium Supplementary Figure S26	Very high (Dragan et al., 2010)	dsDNA (Dragan et al., 2010)
**Group 4: Very high inhibition**						
TOTO 1	Very high	–	−⁣−	Low^3^ Supplementary Figure S26	Very high (Haugland, 2010)	dsDNA/ssDNA (Haugland, 2010)
SYTO 24	Very high	–	−⁣−	High^3^ (Radvanszky et al., 2015) Supplementary Figure S26)	Low (Haugland, 2010)	dsDNA/ssDNA (Erve et al., 2006; Haugland, 2010)
SYBR Gold	Very high	–	−⁣−	High^3^ Supplementary Figure S26	High (Milanovich et al., 1996)	Sensitive with dsDNA/ssDNA (Haugland, 2010; Tuma et al., 1999)

*T_*m*_ shift: Low (0–3°C), medium (3–6°C) and high (above 6°C). ^1^Optimal dye concentration experiment was performed in the presence of 2 ng DNA per reaction; ^2^Reference; ^3^This study.*

**Group 1** – **non-inhibition** includes SYTO 9, SYTO 13, SYTO 16, SYTO 64, SYTO 82, Boxto, Miami Green, Miami Yellow, and Miami Orange dyes. In this group, the real-time LAMP amplifications were observed with dye concentrations ranging from 0.5 to 10 μM (Figure 1). These dyes have very small slope values varying from −0.41 ± 0.14 to −0.01 ± 0.04 (Table 2) indicating no inhibitory effect on the efficiency of the real-time LAMP assay. The T_t_ values obtained from the real-time LAMP using SYTO 9, SYTO 13, SYTO 16, Miami Yellow, and SYTO 82, were constant at the dye concentrations of 2–10 μM. Moreover, the constant T_t_ values of the real-time LAMP were also observed with the dye concentrations ranging from 4 to 10 μM for Boxto, Miami Orange, Miami Green and SYTO 64 (Table 1 and Supplementary Figures S1–S9). These concentrations were considered as optimal concentrations of the respective dyes for the real-time LAMP reaction. In Group 1, the shortest T_t_ value of 9.24 ± 0.26 min was observed when using SYTO 9 at an optimal concentration. Similarly, short T_t_ values were also achieved when using other dyes such as SYTO 64 (9.62 ± 0.21 min), SYTO 16 (9.88 ± 0.23 min), SYTO 82 (10.20 ± 0.29 min), SYTO 13 (11.01 ± 0.01 min), Boxto (11.04 ± 0.22 min), Miami Orange (11.67 ± 0.30 min), Miami Yellow (11.47 ± 0.35 min) and Miami Green (13.67 ± 0.30 min) (Table 1).

**TABLE 2:** Dyes grouped based on the slopes of each dye in real-time LAMP reactions.

**Dye/Group**	**Slope (Figure 1)**
**Group 1: No inhibition**	
Miami Orange	−0.41 ± 0.14
Miami Green	−0.39 ± 0.18
Boxto	−0.35 ± 0.26
Miami Yellow	−0.32 ± 0.13
SYTO 64	−0.22 ± 0.07
SYTO 9	−0.13 ± 0.09
SYTO 82	−0.04 ± 0.08
SYTO 16	−0.04 ± 0.07
SYTO 13	−0.01 ± 0.04
**Group 2: Medium inhibition**	
YOPRO 1	1.88 ± 0.53
SYTO 62	2.17 ± 1.22
TOPRO 3	3.02 ± 0.90
Eva Green	7.30 ± 1.21
SYTO 60	7.41 ± 1.33
**Group 3: High inhibition**	
NG-DCS1	12.17 ± 1.03
POPO 3	14.81 ± 2.15
TOTO 3	17.09 ± 1.07
SYBR Green I	25.42 ± 5.68
Pico 488	52.99
BOBO 3	71.6

**Group 2** including YOPRO 1, SYTO 62, TOPRO 3, SYTO 60 and Eva Green presents a **medium inhibitory** effect on the real-time LAMP assay. The T_t_ values of the real-time LAMP when using these dyes increased gradually when the concentrations of the dye were increased from 0.5× to 5× or 0.5 to 5 μM, and a total inhibition effect on the real-time LAMP assay was observed at 10 μM (or 10×) concentration (Figure 1 and Supplementary Figures S10–S14). Moreover, the use of these dyes in the real-time LAMP reactions generated bigger slope values than the dyes in Group 1. The slope values varied from 1.88 ± 0.53 to 7.41 ± 1.33 indicating a medium inhibitory effect of these dyes on the efficiency of the real-time LAMP assay (Table 2). The T_t_ values were 10.97 ± 0.17, 11.15 ± 0.19, 11.94 ± 0.23, 14.84 ± 0.38, and 15.83 ± 0.09 for TOPRO 3, SYTO 60, SYTO 62, YOPRO 1, and Eva Green, respectively, at an optimal concentration (Table 1).

POPO3, DCS1, SYBR Green I, BOBO 3, Pico 488, and TOTO 3 had a **high inhibitory** effect. When using these dyes in the real-time LAMP reactions at a dye concentration above 3 μM, a total inhibition was observed (Figure 1 and Supplementary Figures S15–S20). The plots of the T_t_ against the dye concentrations revealed very steep slopes ranging from 12.17 ± 1.03 to 71.6 (Table 2). These dyes were therefore classified in **Group 3** as dyes with a high inhibitory effect on the real-time LAMP (Table 1). The optimal dye concentrations for the real-time LAMP of this group were very low and were determined to be 0.5× for SYBR Green I and 0.5 μM for all the other dyes in this group. The T_t_ values at optimal concentration were 9.27 ± 0.18 min for DCS1; 14.24 ± 0.54 min for TOTO3, 15.22 ± 0.23 min for SYBR Green I; 15.67 ± 0.38 min for POPO 3, 16.49 ± 0.41 for Pico 488, and 17.34 ± 0.40 min for BOBO 3 (Table 1).

When using TOTO 1, SYTO 24, and SYBR Gold (**Group 4**) in the real-time LAMP assay, the fluorescence signal was observed only at the lowest dye concentration tested, i.e., 0.5 μM (TOTO 1 and SYTO 24) or 0.5× (SYBR Gold). From 1 μM (or 1×) concentration and higher, no fluorescence signal was recorded for dyes in this group. Besides, the fluorescence signal at 0.5 μM or 0.5× was very low (Supplementary Figures S21–S23 in Supplementary Data). Results of gel electrophoresis analysis coincided with real-time fluorescence measurement as no LAMP product was observed with the dye concentration from 1 μM (or 1×) to 10 μM (or 10×), accept 0.5 μM (or 0.5×). These dyes in Group 4 were therefore classified as having **very high inhibitory** effect.

### Fluorescence Intensity During the Real-Time LAMP

**FIGURE 2:**
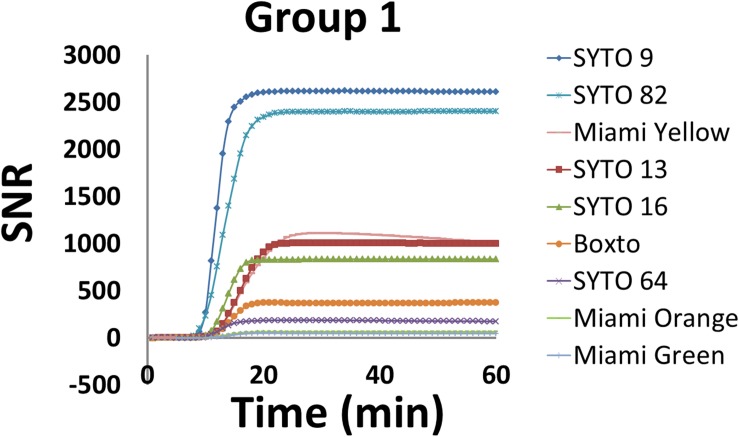
Signal-to-noise ratio (SNR) of nine dyes in Group 1 which showed no inhibition effect. The reaction was performed at 5 μM dye concentration in the presence of 2 ng of *S.* Enteritidis DNA per test.

Signal to noise ratio is an important characteristic to monitor real-time DNA amplification (real-time PCR and real-time LAMP). In this study, we therefore calculated SNR according to equation 2 (eq. 2) and evaluated the SNR for the nine dyes in Group 1 which showed no inhibition effect on the real-time LAMP assay. Figure 2 shows the SNR of these dyes in the real-time LAMP reactions. These real-time LAMP assays were performed at 5 μM dye concentration in the presence of 2 ng of genomic *S.* Enteritidis. Of the nine dyes tested, SYTO 9 showed the highest SNR with a maximum SNR (SNR_max_) of 2611 times the noise level, while SYTO 82 showed a SNR_max_ of 2403. SYTO 13, SYTO 16 and Miami Yellow showed SNR_max_ between 835 and 1108 (Figure 2). In contrast, the SNR_max_ of Boxto, SYTO 64, Miami Orange and Miami Green were lower ranging from 49 to 372. The lowest SNR_max_ were observed from Miami Orange and Miami Green with SNR_max_ of 64 and 49, respectively. In addition, the SNRs of some of the dyes in Groups 2 and 3 were also determined at optimal concentration. The SNR_max_ of these dyes were lower than that of SYTO 9, SYTO 82, SYTO 13, SYTO 16 and Miami Yellow in group 1, ranging between 52 and 581 (Supplementary Figure S24).

### Investigation of the Sensitivity of Real-Time LAMP

**FIGURE 3:**
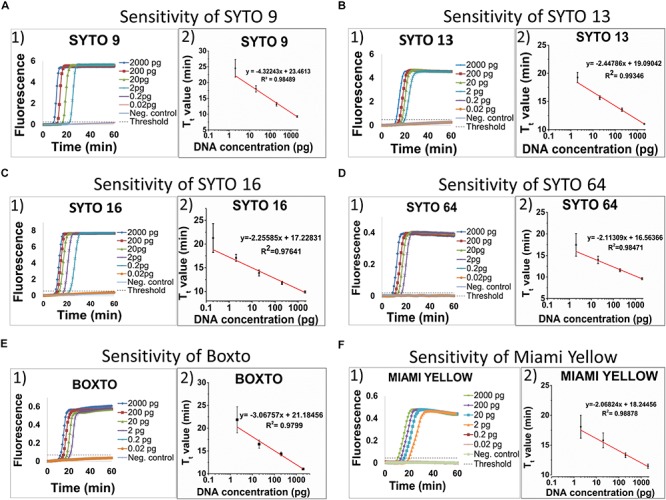
Sensitivity of six dyes in Group 1 including SYTO 9, SYTO 13, SYTO 16, SYTO 64, Boxto and Miami Yellow, which had no inhibitory effect and SNR above 100. The sensitivity test was performed at 5 μM dye concentration for each dye. **A–F** (from the left to the right): (1) Raw fluorescence signal (arbitrary unit), and (2) Standard curves.

The limit of detection (LOD) of the real-time LAMP based on the amplification of the *hil*A gene for detection of *Salmonella* spp. was used to investigate and to compare the use of different dyes in the 3 groups (1, 2, and 3). Figure 3 shows the sensitivity of the 6 dyes from Group 1 in the real-time LAMP assays. When using SYTO 9 (Figure 3A1), SYTO 13 (Figure 3B1), SYTO 64 (Figure 3D1), Boxto (Figure 3E1) and Miami Yellow (Figure 3F1), a similar sensitivity of the real-time LAMP assay was observed with LOD = 2 pg (equivalent to 386 copies) of *Salmonella* genome per reaction in a short time (T_t_ = 17.46 ± 2.63 min for SYTO 64, T_t_ = 18.08 ± 1.93 for Miami Yellow, T_t_ = 19.26 ± 0.91 min for SYTO 13, T_t_ = 21.85 ± 2.87 min for Boxto and T_t_ = 24.51 ± 2.79 min for SYTO 9) at 65°C (Figures 3A,B,D,F). Interestingly, a sensitivity 10-fold better than that of all the other dyes was achieved when using SYTO 16 (Figure 3C) with LOD = 0.2 pg (equivalent to 38 copies) and with T_t_ = 9.88 ± 0.23 and T_t_ = 21.25 ± 3.06 min for template concentrations of 2 and 0.2 pg, respectively (Figure 3C). We expected that all the dyes in Group 2 (medium inhibition) and Group 3 (high inhibition) could influence the sensitivity of real-time LAMP detection. Table 3 shows the LOD of the real-time LAMP when using the dyes in these two groups. As expected, when using these dyes, the LOD was 10–100 times higher than that of the dyes in Group 1 (no inhibition) (Supplementary Figure S25). Moreover, when plotting four or five different concentrations of DNA targets per reaction against the T_t_ values, a good linear relationship was obtained (Figures 3A2–F2 and Supplementary Figure S25). When the DNA template concentrations per reaction ranging from 2000 to 2 pg were used, the T_t_ values ranging from 9.24 ± 0.26 to 24.51 ± 2.79 min for SYTO 9, 9.88 ± 0.23 to 21.25 ± 3.06 min for SYTO 16; 11.01 ± 0.01 to 19.26 ± 0.91 min for SYTO 13, 9.62 ± 0.21 to 17.46 ± 2.63 min for SYTO 64, 11.04 ± 0.22 to 21.85 ± 2.87 min for BOXTO, and 11.47 ± 0.35 to 18.08 ± 1.93 min for Miami

**TABLE 3:** The limit of detection (LOD) per reaction and doubling time (DT) of different dyes tested in this study at its optimal concentration.

**Dye/group**	**Optimal dye concentration**	**LOD**	**DT (min)**
**Group 1**			
SYTO 9	5 μM	2 pg	1.301181
SYTO 13	5 μM	2 pg	0.736879
SYTO 16	5 μM	0.2 pg	0.679079
SYTO 64	5 μM	2 pg	0.636103
Boxto	5 μM	2 pg	0.923431
Miami Yellow	5 μM	2 pg	0.622603
**Group 2**			
SYTO 62	2 μM	20 pg	0.614553
TOPRO 3	2 μM	20 pg	0.698236
SYTO 60	2 μM	20 pg	0.714618
EvaGreen	1×	20 pg	0.742951
**Group 3**			
SYBR Green I	0.5×	20 pg	0.660604
DCS1	0.5 μM	20 pg	0.697222

Yellow were determined (Figures 3A–F). In addition, to estimate the speed of the real-time LAMP reaction, a DNA doubling time (DT) was determined using equation 3, based on the T_t_ value against concentrations of the *S.* Enteritidis DNA template in the reaction for each dye. Lower DT values ranging from 0.62 to 0.73 min (when using SYTO 16, SYTO 13, SYTO 64, and Miami Yellow at 5 μM) in comparison to 1.30 min for SYTO 9 and 0.92 min for BOXTO were determined (Table 3). Similar DT values were obtained with two other dye groups (Groups 2 and 3) at a higher DNA concentration (20 pg) when using the dye concentration of 2 μM (Table 3).

### Comparison of SYTO 9 in Different LAMP Reactions and Detection Systems

**FIGURE 4:**
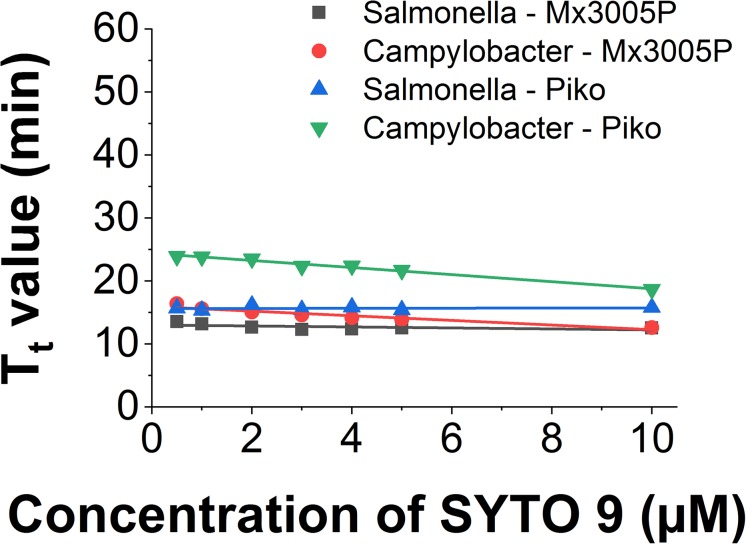
Comparison of T_t_ value against dye concentration of SYTO 9 in different LAMP reactions and different detector systems.

The use of SYTO 9 in different LAMP reactions and different systems showed slight differences in T_t_ value. In the Mx3005P system, the T_t_ of *Salmonella* LAMP reaction is shorter (2.8 min) than for the *Campylobacer* LAMP reaction at a low dye concentration of 0.5 μM. However, when increasing the dye concentration, the T_t_ values of the two LAMP reactions became closer and almost similar at 10 μM concentration of SYTO 9. The T_t_ values of *Salmonella* and *Campylobacter* LAMP reactions behaved in the same manner in the Piko system. Nevertheless, the gaps of T_t_ values between the two LAMP reactions from low (0.5 μM) to high (10 μM) concentration of SYTO 9 in the Piko system (8.2 to 2.8 min, respectively) were bigger as compared to the Mx3005P (2.8 to 0 min, respectively) (Figure 4).

Similarly, the small change of slope was also observed when using the same LAMP amplicon in different systems (Figure 4). The slope value of the *Salmonella* LAMP reaction was −0.13 ± 0.09 in the Chromo4 system, −0.08 ± 0.05 in the Mx3005P system, and 0.01 ± 0.04 in the Piko system. In the *Campylobacter* LAMP reaction, the slope values were −0.56 ± 0.03 and −0.37 ± 0.05 using Piko and Mx3005P system, respectively (Table 4). Although there was a variation of slope values in the three tested systems, the change was very small. Comparing to the slope values of all dyes tested in this study, the slope values of SYTO 9 using *Salmonella* LAMP reaction (−0.13 to 0.01) and *Campylobacter* LAMP reaction (−0.56 to −0.37) are still classified in Group 1 without inhibition effect to the LAMP reaction. Furthermore, the melting curves of the two LAMP reactions were analyzed in the Piko system and the results showed that the melting temperature was almost similar (83°C for *Salmonella* amplicon and 80°C for *Campylobacter* amplicon) with dye concentrations from 0.5 to 10 μM (Supplementary Figure S27). This result also indicated that SYTO 9 has no inhibition effect on the LAMP reaction.

**TABLE 4:** The slopes of different LAMP reactions and different detection systems.

**Amplicons and systems**	**Slope**
Salmonella–Mx3005P	−0.08 ± 0.05
Salmonella–Piko	0.01 ± 0.04
Salmonella–Chromo4	−0.13 ± 0.09
Campylobacter–Mx3005 P	−0.37 ± 0.05
Campylobacter–Piko	−0.56 ± 0.03

In contrast, detection limits of fluorescence were significantly different between the three tested systems. In the Mx3005P system, the saturation of fluorescence was observed at 3 μM concentration of SYTO 9 and higher (Supplementary Figure S28) while saturation was not observed at any of the tested concentrations ranging from 0.5 to 10 μM in the Chromo4 (Supplementary Figure S1) and Piko machines (Supplementary Figure S29).

## Discussion

We have investigated a panel of 23 DNA dyes belong to four groups of dyes with different optical properties for use in real-time LAMP. By analyzing the T_t_ values against the dye concentrations in the real-time LAMP reaction in combination with gel electrophoresis analysis, we categorized the tested dyes into four groups: (1) non-inhibition effect; (2) medium inhibition effect; (3) high inhibition effect; and (4) very high inhibition effect. The nine dyes in Group 1 (non-inhibition effect) showed real-time LAMP amplification in a wide range of dye concentrations (0.5–10 μM) with very small slope values (−0.41 ± 0.14 to −0.01 ± 0.04). Among these nine dyes, the shortest T_t_ value of 9.24 ± 0.26 min was observed for SYTO 9. However, testing the sensitivity of the real-time LAMP reaction revealed that when using SYTO 16 a tenfold lower LOD was achieved within 21.25 ± 3.06 min. Therefore, for development of a rapid real-time LAMP detection SYTO 9 is the best candidate, while SYTO 16 should be used for a highly sensitive real-time LAMP.

Gudnason et al. (2007) studied the effects of dye concentration, sequence composition of DNA amplification and melting temperature testing several different DNA dyes for real-time PCR. Results from this study suggest that the inhibition mechanism of dyes on an increase in cycle threshold (C_t_) value may depend on the binding affinity of each dye to dsDNA (Gudnason et al., 2007). Similarly, the increase in T_t_ that we observed could be due to a high binding affinity of the dye to the DNA and thereby stabilizing the dsDNA and hindering the strand displacement activity of the Bst DNA polymerase in the real-time LAMP amplification. The dyes in Group 1 including SYTO 9, SYTO 13, SYTO 16, SYTO 64, SYTO 82 and Boxto have low binding affinity (Eriksson, 2003; Haugland, 2010) and this could explain why they have no inhibition effect on the real-time LAMP reaction (There is no information on binding affinity for the Miami Green, Miami Yellow, and Miami Orange dyes) (Table 1). In contrast, dyes with higher binding affinity such as YOPRO 1, TOPRO 3 (Group 2) and Nuclear Green, SYBR Green (Group 3), SYTO 24, and SYBR Gold (Group 4) (Mao et al., 2007; Haugland, 2010; Kirsanov et al., 2010; AAT Bioquest, 2016) inhibited the real-time LAMP amplification (Table 1). The tightness of the dye binding to the DNA was also suggested as a reason for the increase in C_t_ that Gudnason et al. (2007) observed as this could stabilize the dsDNA and negatively influence the extension of the new strand (Gudnason et al., 2007). The tightness of the dye binding was evaluated through melting curve analysis. For some of the dyes, the melting temperature (T_m_) of the PCR amplicon increased with increasing dye concentrations, whereas for others the dye concentration had little or no influence on the T_m_. The shift of the T_m_ value i.e., the difference in T_m_ between low and high dye concentrations indicated the dye’s stabilizing effect on dsDNA. The higher T_m_ shift, the stronger the stabilization effect of the dye on dsDNA. Most of the dyes in Group 1 including the dyes in Miami groups showed a low maximum T_m_ shift (Gudnason et al., 2007; Supplementary Figure S26) and no inhibition effect in a wide range of dye concentrations (2–10 μM) (Table 1). Dyes in Group 2 (YOPRO 1, SYTO 62, TOPRO 3, and SYTO 60) showed a medium maximum T_m_ shift (Gudnason et al., 2007) and medium inhibition effect in a range of dye concentrations from 2–10 μM (Table 1). Another possibility to explain the inhibition effect of a dye could be the binding affinity of the dye to single-strand DNA (ssDNA) which may be more dominant than binding to dsDNA (Mao et al., 2007). In this case, dyes with higher binding affinity to ssDNA may bind to primers and loop primers in the real-time LAMP reaction, which may prevent the annealing of the primers to the DNA template and hinder the activity of *Bst* DNA polymerase as well as interfere with the extension step. For most of the dyes, the inhibition effect on real-time LAMP amplification could be explained as a combination of high binding affinity to ds/ssDNA and/or high stabilizing effect on dsDNA as indicated by the high shift in T_m_ (Table 1).

In Group 1 all the dyes both had a low shift in T_m_ (low stabilizing effect on dsDNA) and a low binding affinity for ds/ssDNA and these dyes had no inhibition effects on the LAMP reaction (Table 1).

In Group 2 most of the dyes (YOPRO 1, SYTO 62, TOPRO 3, SYTO 60) caused a medium shift in T_m_. In contrast, Eva Green showed low maximum T_m_ shift (Supplementary Figure S26). The DNA binding affinity of Eva Green is lower than that of SYBR Green I. However, it is known that the DNA binding affinity for SYBR Green I is very high, so the binding affinity of Eva Green may still be sufficiently high to explain the medium inhibition effect observed for this dye (Table 1). Alternatively, Eva Green could be an exception to the general picture observed for all the other dyes.

In Group 3, Nuclear Green DCS1 and SYBR Green I showed high or very high binding affinity to dsDNA (Haugland, 2010; AAT Bioquest, 2016) and high maximum T_m_ shift to dsDNA (Gudnason et al., 2007; Radvanszky et al., 2015; Supplementary Figure S26), and therefore these dyes may stabilize the dsDNA resulting in a high inhibitory effect to the real-time LAMP (Table 1). In contrast, three other dyes in this group; the TOTO 3, POPO 3, and BOBO 3 showed a low maximum T_m_ shift of dsDNA (Gudnason et al., 2007) but have a very high binding affinity to DNA (Haugland, 2010). So far no data on the preferable binding to dsDNA in comparison to ssDNA of these three dyes has been reported. However, the low T_m_ shift in combination with a high DNA binding affinity indicates that these dyes could bind more strongly to ssDNA. In the LAMP reaction, the available free ssDNA are primers and loop positions, and strong binding of dye here could prevent the synthesis of new DNA strand in the real-time LAMP reaction and thereby inhibit the reaction. This could explain the high inhibitory effects observed when using these dyes in the real-time LAMP reaction.

In Group 4, all the dyes tested showed a very high inhibition effect (Supplementary Figures S21–S23). The three dyes in this group (TOTO 1; SYTO 24, and SYBR gold) showed a capacity to bind to both dsDNA and ssDNA at different levels (Tuma et al., 1999; Erve et al., 2006; Haugland, 2010; Kirsanov et al., 2010). However, the dyes also show different characteristics such as SYTO 24 has a low binding affinity to DNA while both SYBR Gold and TOTO 1 have high or very high binding affinity to DNA (Haugland, 2010; Kirsanov et al., 2010). In addition, both SYTO 24 and SYBR gold have a very high maximum T_m_ shift to dsDNA (Radvanszky et al., 2015; Supplementary Figure S26) while TOTO 1 has very low T_m_ shift (Table 1 and Supplementary Figure S26). Therefore one possible explanation for the very high inhibition effects for the dyes in this group is the capacity of the dyes in this group to bind to both the template (dsDNA) and the primers (ssDNA); as a consequence this binding activity prevent the extension of new strand the real-time LAMP reaction (Table 1).

We suggest that the DNA dyes have a similar effect when used to detect DNA in a normal LAMP reaction and when detecting RNA targets in reverse transcription LAMP (rtLAMP). Eva Green (Ocwieja et al., 2015) was demonstrated in real-time rtLAMP detection. In other work, we also used SYTO 9 for the detection of the RNA virus Avian Influenza Virus (AIV) and we did not see different behavior comparing with DNA targets (data not shown).

Experiments to optimize the dye concentration in real-time LAMP revealed that the use of optimal dye concentration is an important factor in real-time LAMP detection. For the dyes with inhibition effects, the use of a dye concentration higher than the optimal concentration led to the result that the T_t_ was delayed due to the inhibitory effect of the dyes (Supplementary Figures S10–S20). Moreover, when using dye concentration lower than the optimal concentration for the dyes without inhibition effects, the T_t_ was also delayed as illustrated in this study (Supplementary Figures S1, S4–S9). For example, in the Chromo4 machine, when using different concentrations of SYTO 9 for real-time LAMP (ranging from 0.5 to 10 μM), the shortest T_t_ value of 9.24 min was observed at an optimal dye concentration of 2–10 μM. While the T_t_ increased up to 13 min when a lower dye concentration of 1 μM was used. However, the selection of dye concentration also depends on the detector of each system. E.g., in the Mx3005P system, 1–2 μM of SYTO 9 should be used since it resulted in the shortest T_t_ values and no saturation was observed (Supplementary Figure S28). While in the Piko system, a high concentration such as 5 to 10 μM should be used due to a high fluorescence signal and the shortest T_t_ recorded (Supplementary Figure S29). In POC systems, the detectors are often small, simple and cheap with a resulting limitation of fluorescence detection. Therefore, these systems may need higher dye concentrations than lab-bench detector system. Besides, high background fluorescence should be taken into account when using high dye concentration (5–10 μM) (Supplementary Figure S30). Moreover, in POC assays, the stability of dyes in solution should be considered. Most of the stocks of dyes in this study are in DMSO (except Eva Green in water) which is stable for at least one year at −20°C. However, when diluting in aqueous solution, the stability of the dye is reduced and need to be observed for each dye. BOXTO is recommended to be diluted in MilliQ water and the dye is stable in 4°C for a month (Tataa Biocenter, 2018). Eva Green stock is 20× in water which is claimed to be extremely stable both thermally and hydrolytically (Biotium, 2019). Another study has also reported that SYTO 9 and SYBR Green I were stable in PCR master mixture for 2–3 weeks (Monis et al., 2005).

Following an extensive screening of 23 fluorescent intercalating DNA dyes we have found that SYTO 9, SYTO 13, SYTO 16, SYTO 64, SYTO 82, Boxto, Miami Yellow, Miami Orange and Miami Green (Group 1) are suitable for real-time LAMP. Using these dyes non-inhibitory effects, high sensitivity and good efficiency were achieved. To our best knowledge, this is the first study to investigate the inhibition effect of all these dyes systematically for real-time LAMP reaction. Among these dyes, SYTO 9 showed non-inhibitory effect, maximal SNR, high sensitivity and was not only considered as one of the good candidates suitable for the real-time LAMP amplification, but also in real-time PCR (Monis et al., 2005; Gudnason et al., 2007). However, concerning the speed of the real-time LAMP for rapid detection, SYTO 9 was not a perfect candidate since doubling time of real-time LAMP using SYTO 9 was two times higher than that of all the dyes tested in Group 1 except Boxto (1.4 times). Besides, SYTO 16 was also considered a good candidate for rapid real-time LAMP amplification. Although SYTO 16 showed intermediate SNR, it has no inhibitory effect, low DT and lowest LOD in all the dyes tested. Therefore, with the information of inhibitory effect, SNR, LOD, and DT of the 23 dyes provided by this study, users can consider selecting a dye suitable for their application in real-time LAMP.

In conclusion, SYTO 9, SYTO 82, SYTO 13, SYTO 16 and Miami Yellow were found to have superior properties such as no inhibitory effect, high SNR, and high sensitivity in real-time LAMP assay. These dyes are therefore suitable candidates to apply for the development of new real-time LAMPs and for integrating the real-time LAMP technology in POC systems for rapid online or at site detection of pathogens.

## Data Availability Statement

All datasets generated for this study are included in the manuscript/Supplementary Files.

## Author Contributions

AW, DB, and MM designed the work. TQ and TN performed the experiments. DB, AW, TQ, and TN wrote the manuscript.

## Conflict of Interest

The authors declare that the research was conducted in the absence of any commercial or financial relationships that could be construed as a potential conflict of interest.
