# Supplementary material for: Classification of Multiple DNA Dyes Based on Inhibition Effects on Real-Time Loop-Mediated Isothermal Amplification (LAMP): Prospect for Point of Care Setting
Source: Front Microbiol. 2019 Oct 15;10:2234. doi: 10.3389/fmicb.2019.02234 (PMC6803449; doi:10.3389/fmicb.2019.02234)
Supplement: Supplementary file 1 [file Table_1.docx]

Supplementary Material

Table S1. The primer sequences of HilA gene of *Salmonella* and *Campylobacter* jejuni used in this study.

| **Salmonella primer** | **Sequence (5’-3’)** | **% GC** |
| --- | --- | --- |
| Sal F3 | CGC ATA CTG CGA TAA TCC CTT C | 50 |
| Sal B3 | ATT CTG TCG GAA GAT AAA GAG C | 41 |
| Sal FIP | CGC CGC AAC CTA CGA CTC ATA  CTG CAG ACT CTC GGA TTG AAC C | 55.5 |
| Sal BIP | GGC GGA GAC ACC ACT ACG ACC  GTT ACA TTG AAA CAC TGT ACG | 53 |
| Sal LF | CTT CCT TTT CAG ATG CAG GAT CA | 43 |
| Sal LB | GGA CGA TTA AAC CGA TAG CCC T | 53 |
| LAMP product |  | 53.7 |
| **Campylobacter jejuni primer** | **Sequence (5’-3’)** | **% GC** |
| Cj F3 | GCAAGACAATATTATTGATCGC | 36.4 |
| Cj B3 | CTTTCACAGGCTGCACTT | 50 |
| Cj FIP | ACAGCACCGCCACCTATAGT  AGAAGCTTTTTTAAACTAGGGC | 45.7 |
| Cj BIP | AGGCAGCAGAACTTACGCATT  GAGTTTGAAAAAACATTCTACCTCT | 39.8 |
| Cj LF | CTAGCTGCTACTACAGAACCAC | 50 |
| Cj LB | CATCAAGCTTCACAAGGAAA | 40 |
| LAMP product |  | 41.4 |

| **Dyes** | **Filter setting** | **Ex/Em** |
| --- | --- | --- |
| SYTO 9 | 1 | 485⁄498 |
| SYTO 13 | 1 | 488/509 |
| SYTO 16 | 1 | 488/518 |
| SYTO 64 | 3 | 599/619 |
| SYTO 82 | 2 | 541⁄560 |
| Boxto | 2 | 515/552 |
| Miami Green | 1 | 414/504 |
| Miami Yellow | 2 | 502/558 |
| Miami Orange | 3 | 535/640 |
| YOPRO 1 | 1 | 491/509 |
| SYTO 62 | 4 | 649⁄680 |
| TOPRO 3 | 4 | 642/661 |
| SYTO 60 | 4 | 652⁄678 |
| Eva Green | 1 | 500/530 |
| dsGreen | 1 | 454/524 |
| POPO3 | 2 | 534/570 |
| Nuclear Green DCS1 | 1 | 503/526 |
| SYBR Green I | 1 | 497/520 |
| BOBO3 | 3 | 570/602 |
| TOTO 3 | 4 | 642/660 |
| Pico 488 | 1 | 503/526 |
| TOTO 1 | 1 | 514/533 |
| SYTO 24 | 1 | 490⁄515 |
| SYBR Gold | 1 | 495/537 |

Table S2. Excitation, emission and filter setting of dyes used in this study.

Table S3. Fluorescence excitation and detection ranges of 4 channels used in Chromo4 system.

|  | Fluorescence excitation ranges | Fluorescence detection ranges |
| --- | --- | --- |
| Channel 1 | 450-490 nm | 515-530 nm |
| Channel 2 | 500-535 nm | 560-580 nm |
| Channel 3 | 555-585 nm | 610-650 nm |
| Channel 4 | 620-650 nm | 675-730 nm |

**
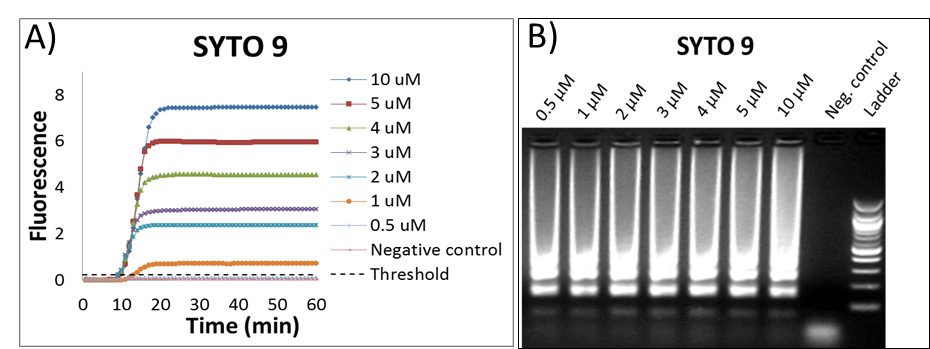
**

Figure S1. Testing of real-time LAMP amplification efficiency of SYTO 9 at different concentrations: A) Raw data of fluorescence intensity recording during LAMP reaction and B) Gel electrophoresis image after LAMP reaction.


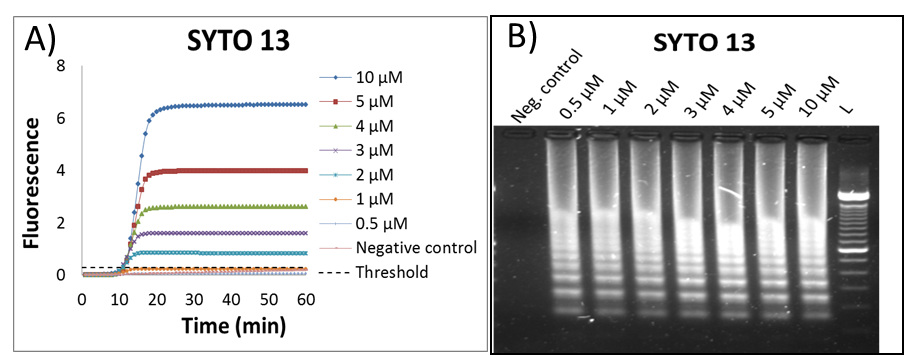


Figure S2. Testing of real-time LAMP amplification efficiency of SYTO 13 at different concentrations: A) Raw data of fluorescence intensity recording during LAMP reaction and B) Gel electrophoresis image after LAMP reaction.


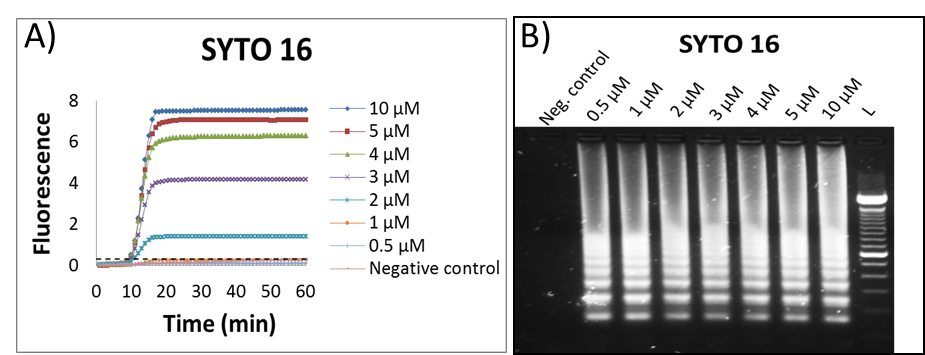


Figure S3. Testing of real-time LAMP amplification efficiency of SYTO 16 at different concentrations: A) Raw data of fluorescence intensity recording during LAMP reaction and B) Gel electrophoresis image after LAMP reaction.


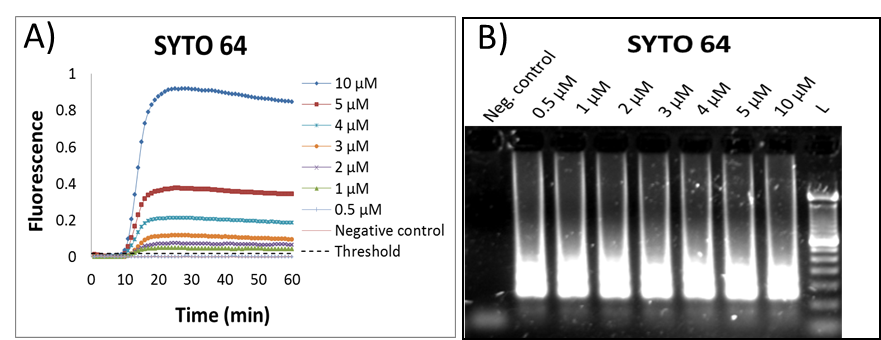


Figure S4. Testing of real-time LAMP amplification efficiency of SYTO 64 at different concentrations: A) Raw data of fluorescence intensity recording during LAMP reaction and B) Gel electrophoresis image after LAMP reaction.


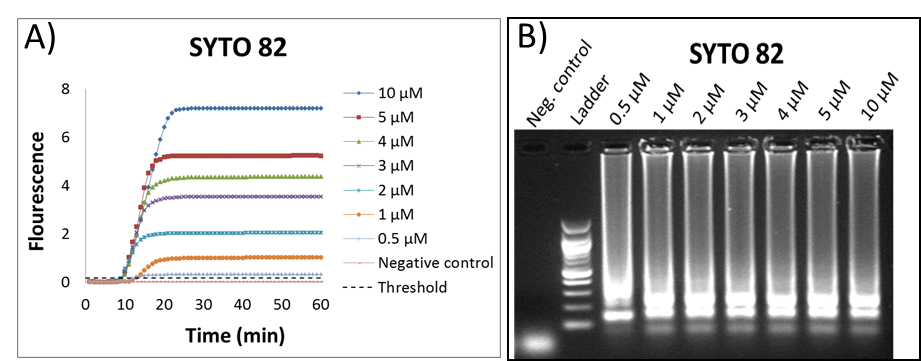


Figure S5. Testing of real-time LAMP amplification efficiency of SYTO 82 at different concentrations: A) Raw data of fluorescence intensity recording during LAMP reaction and B) Gel electrophoresis image after LAMP reaction.


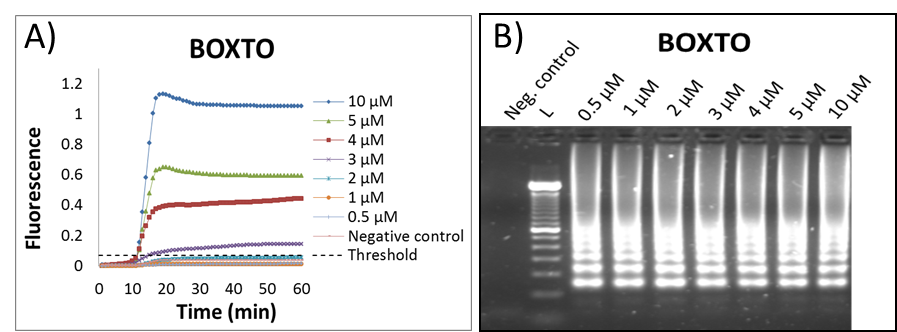


Figure S6. Testing of real-time LAMP amplification efficiency of BOXTO at different concentrations: A) Raw data of fluorescence intensity recording during LAMP reaction and B) Gel electrophoresis image after LAMP reaction.


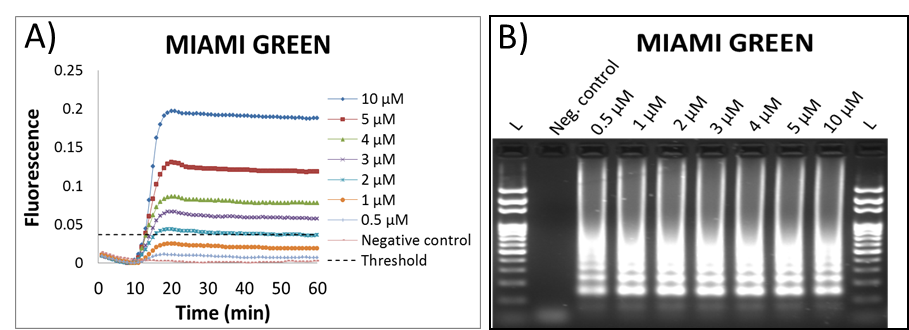


Figure S7. Testing of real-time LAMP amplification efficiency of Miami Green at different concentrations: A) Raw data of fluorescence intensity recording during LAMP reaction and B) Gel electrophoresis image after LAMP reaction.


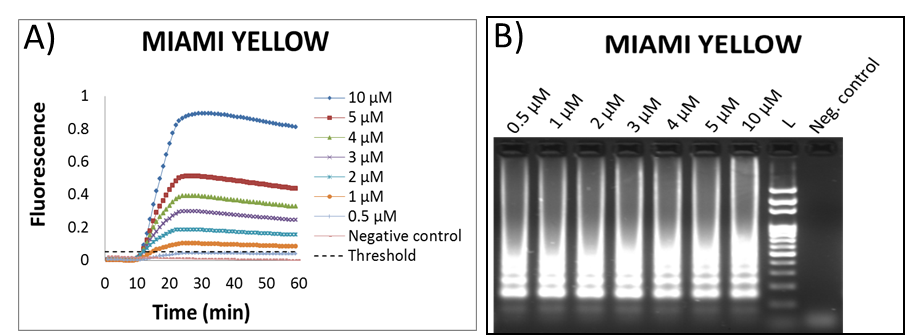


Figure S8. Testing of real-time LAMP amplification efficiency of Miami Yellow at different concentrations: A) Raw data of fluorescence intensity recording during LAMP reaction and B) Gel electrophoresis image after LAMP reaction.


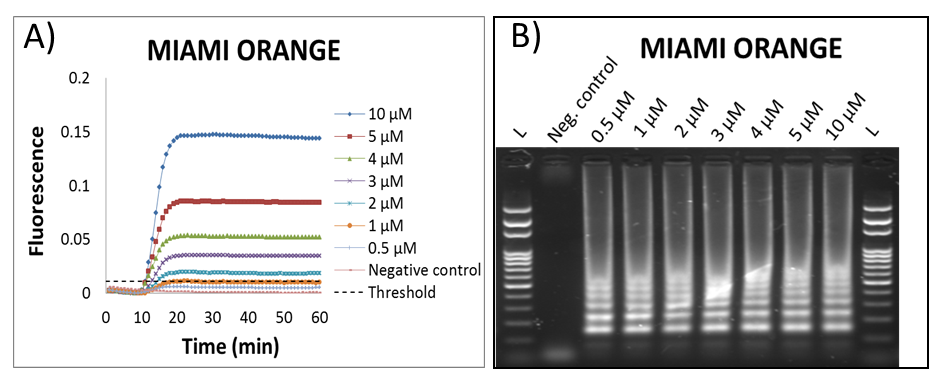


Figure S9. Testing of real-time LAMP amplification efficiency of Miami Orange at different concentrations: A) Raw data of fluorescence intensity recording during LAMP reaction and B) Gel electrophoresis image after LAMP reaction.


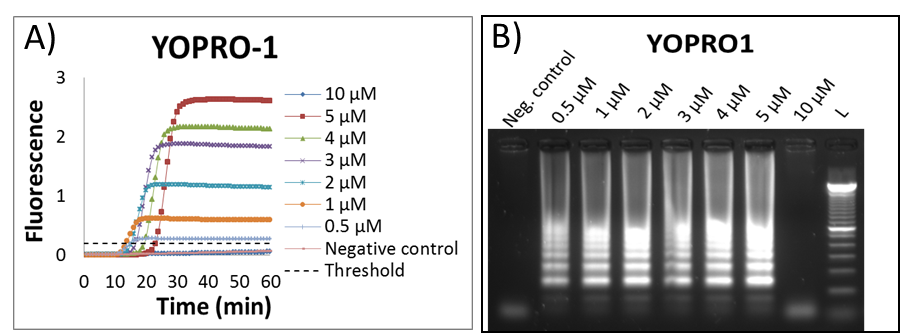


Figure S10. Testing of real-time LAMP amplification efficiency of YOPRO 1 at different concentrations: A) Raw data of fluorescence intensity recording during LAMP reaction and B) Gel electrophoresis image after LAMP reaction.


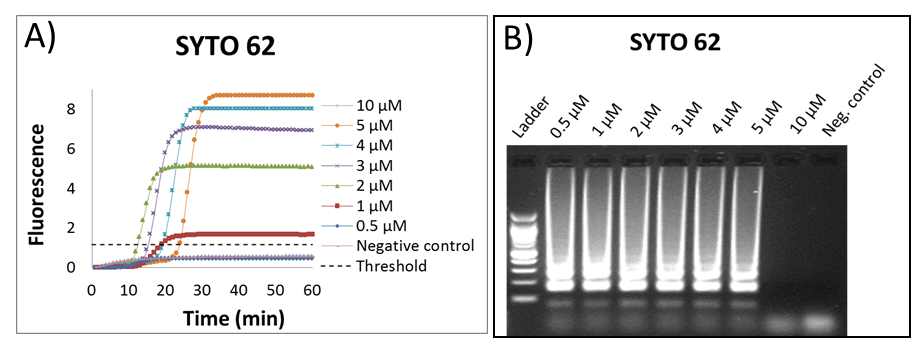


Figure S11. Testing of real-time LAMP amplification efficiency of SYTO 62 at different concentrations: A) Raw data of fluorescence intensity recording during LAMP reaction and B) Gel electrophoresis image after LAMP reaction.


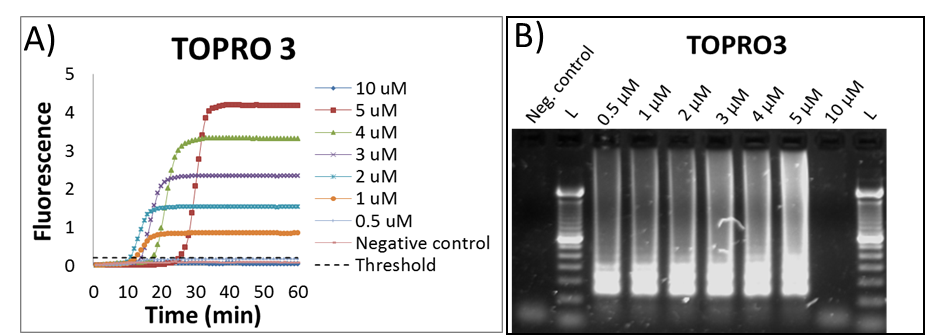


Figure S12. Testing of real-time LAMP amplification efficiency of TOPRO 3 at different concentrations: A) Raw data of fluorescence intensity recording during LAMP reaction and B) Gel electrophoresis image after LAMP reaction.


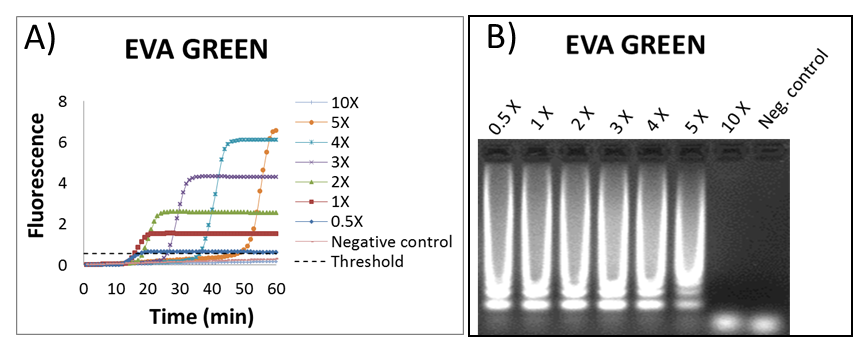


Figure S13. Testing of real-time LAMP amplification efficiency of Eva Green at different concentrations: A) Raw data of fluorescence intensity recording during LAMP reaction and B) Gel electrophoresis image after LAMP reaction.


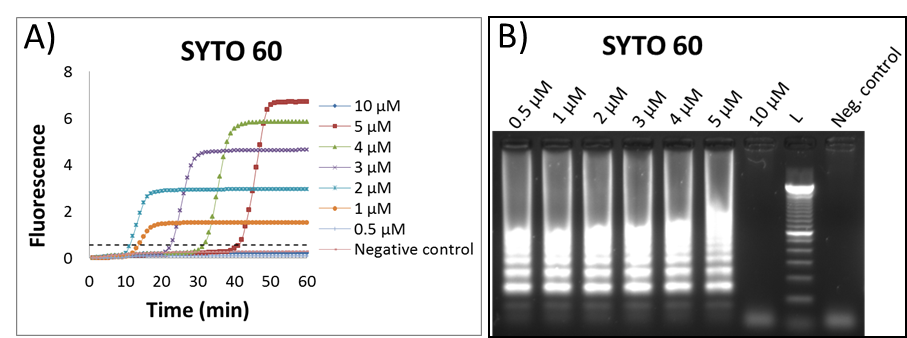


Figure S14. Testing of real-time LAMP amplification efficiency of SYTO 60 at different concentrations: A) Raw data of fluorescence intensity recording during LAMP reaction and B) Gel electrophoresis image after LAMP reaction.


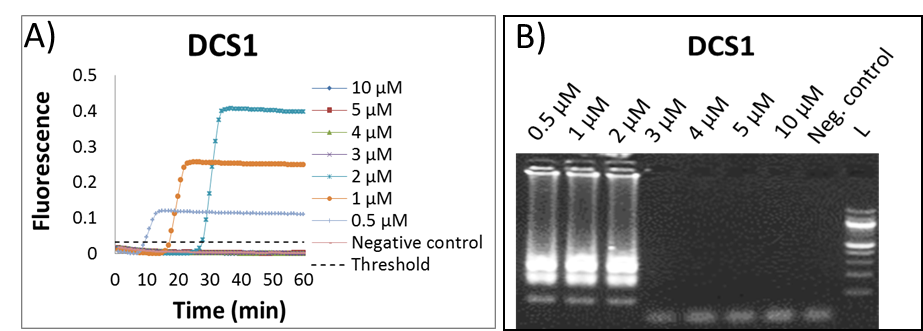


Figure S15. Testing of real-time LAMP amplification efficiency of DCS1 at different concentrations: A) Raw data of fluorescence intensity recording during LAMP reaction and B) Gel electrophoresis image after LAMP reaction.


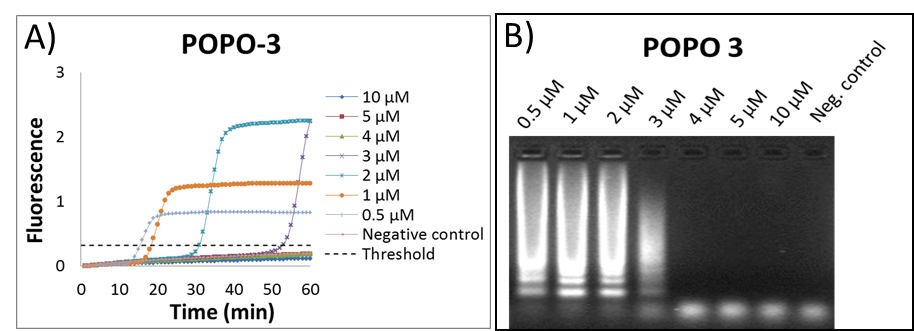


Figure S16. Testing of real-time LAMP amplification efficiency of POPO 3 at different concentrations: A) Raw data of fluorescence intensity recording during LAMP reaction and B) Gel electrophoresis image after LAMP reaction.


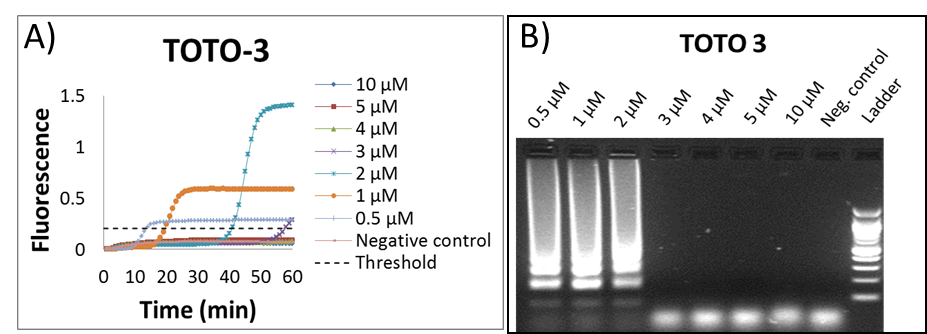


Figure S17. Testing of real-time LAMP amplification efficiency of TOTO 3 at different concentrations: A) Raw data of fluorescence intensity recording during LAMP reaction and B) Gel electrophoresis image after LAMP reaction.


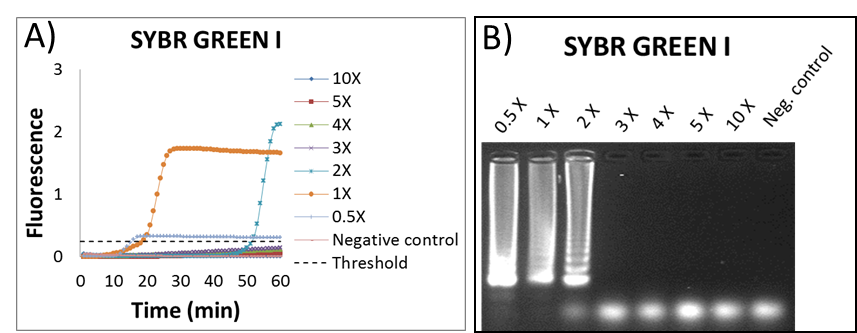


Figure S18. Testing of real-time LAMP amplification efficiency of Sybr Green I at different concentrations: A) Raw data of fluorescence intensity recording during LAMP reaction and B) Gel electrophoresis image after LAMP reaction.


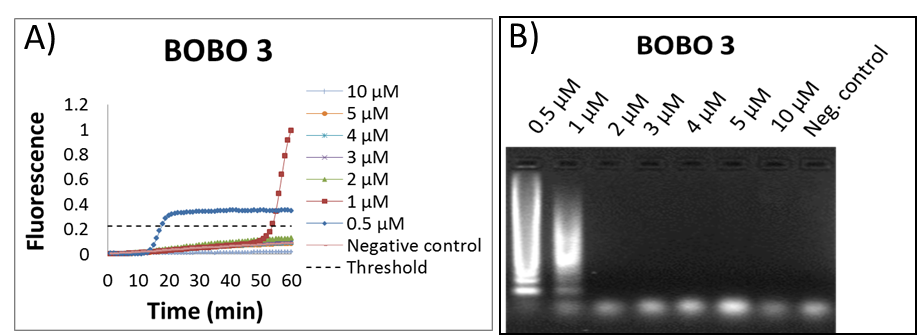


Figure S19. Testing of real-time LAMP amplification efficiency of BOBO3 at different concentrations: A) Raw data of fluorescence intensity recording during LAMP reaction and B) Gel electrophoresis image after LAMP reaction.


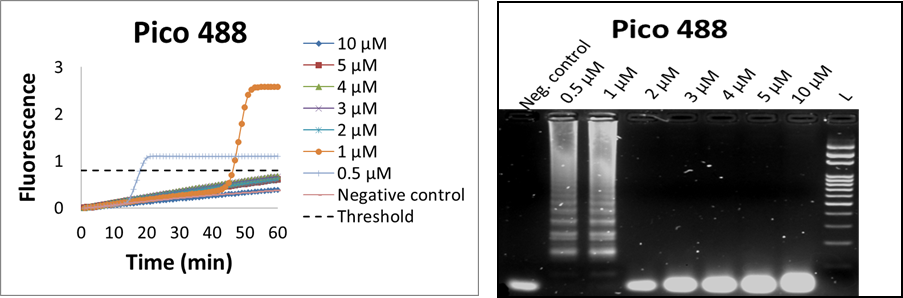


Figure S20. Testing of real-time LAMP amplification efficiency of Pico 488 at different concentrations: A) Raw data of fluorescence intensity recording during LAMP reaction and B) Gel electrophoresis image after LAMP reaction.


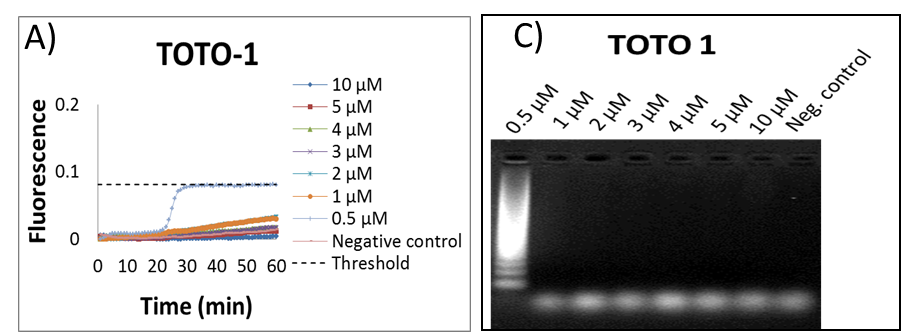


Figure S21. Testing of real-time LAMP amplification efficiency of TOTO 1 at different concentrations: A) Raw data of fluorescence intensity recording during LAMP reaction and B) Gel electrophoresis image after LAMP reaction.


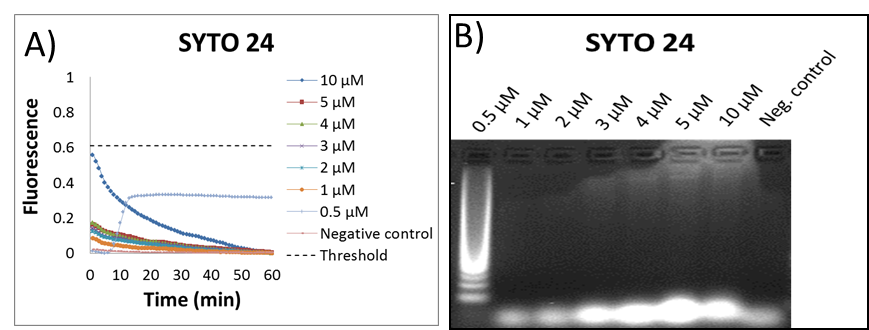


Figure S22. Testing of real-time LAMP amplification efficiency of SYTO 24 at different concentrations: A) Raw data of fluorescence intensity recording during LAMP reaction and B) Gel electrophoresis image after LAMP reaction.


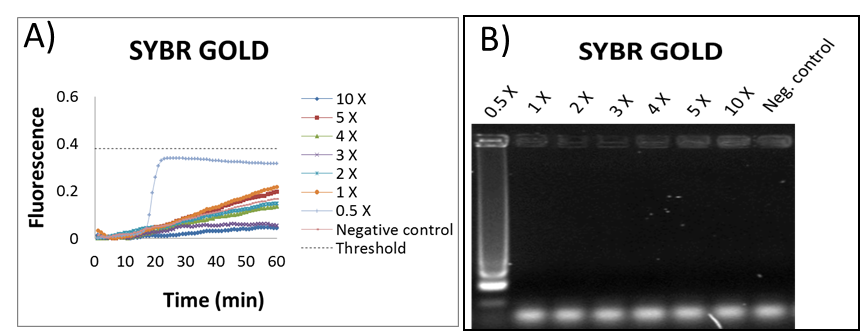


Figure S23. Testing of real-time LAMP amplification efficiency of Sybr Gold at different concentrations: A) Raw data of fluorescence intensity recording during LAMP reaction and B) Gel electrophoresis image after LAMP reaction.


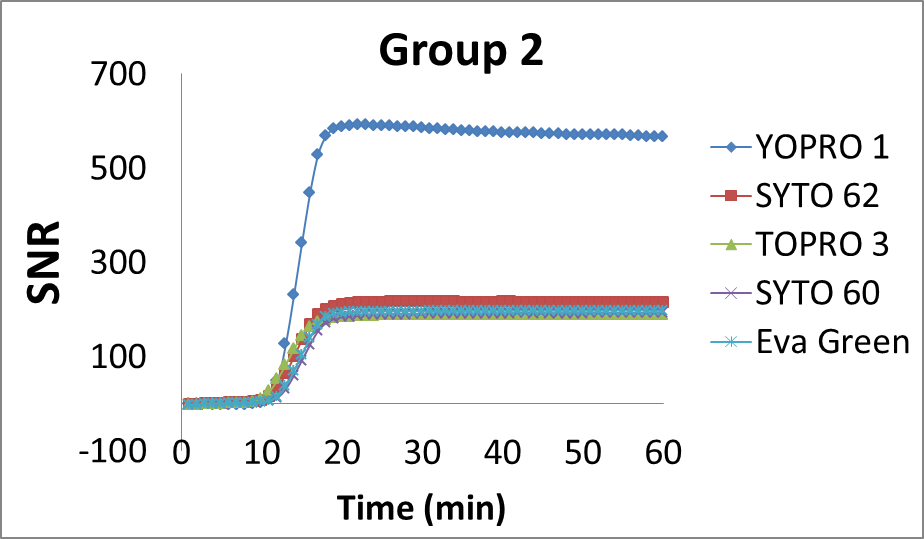

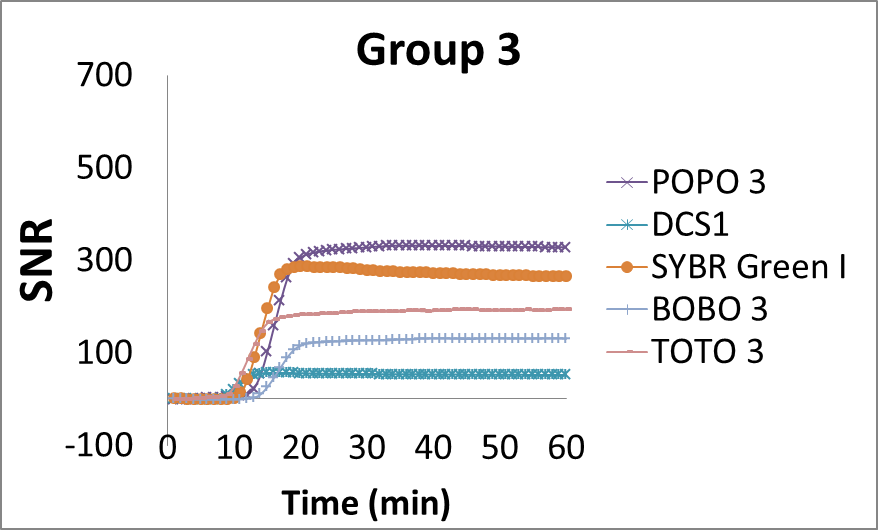


Figure S24. Signal-to-noise ratio of fluorescent dyes in Groups 2 and 3 which showed inhibition effect. The reaction was performed at optimal dye concentration (Table 3) in the presence of 2 ng of *S*. Enteritidis DNA per test.


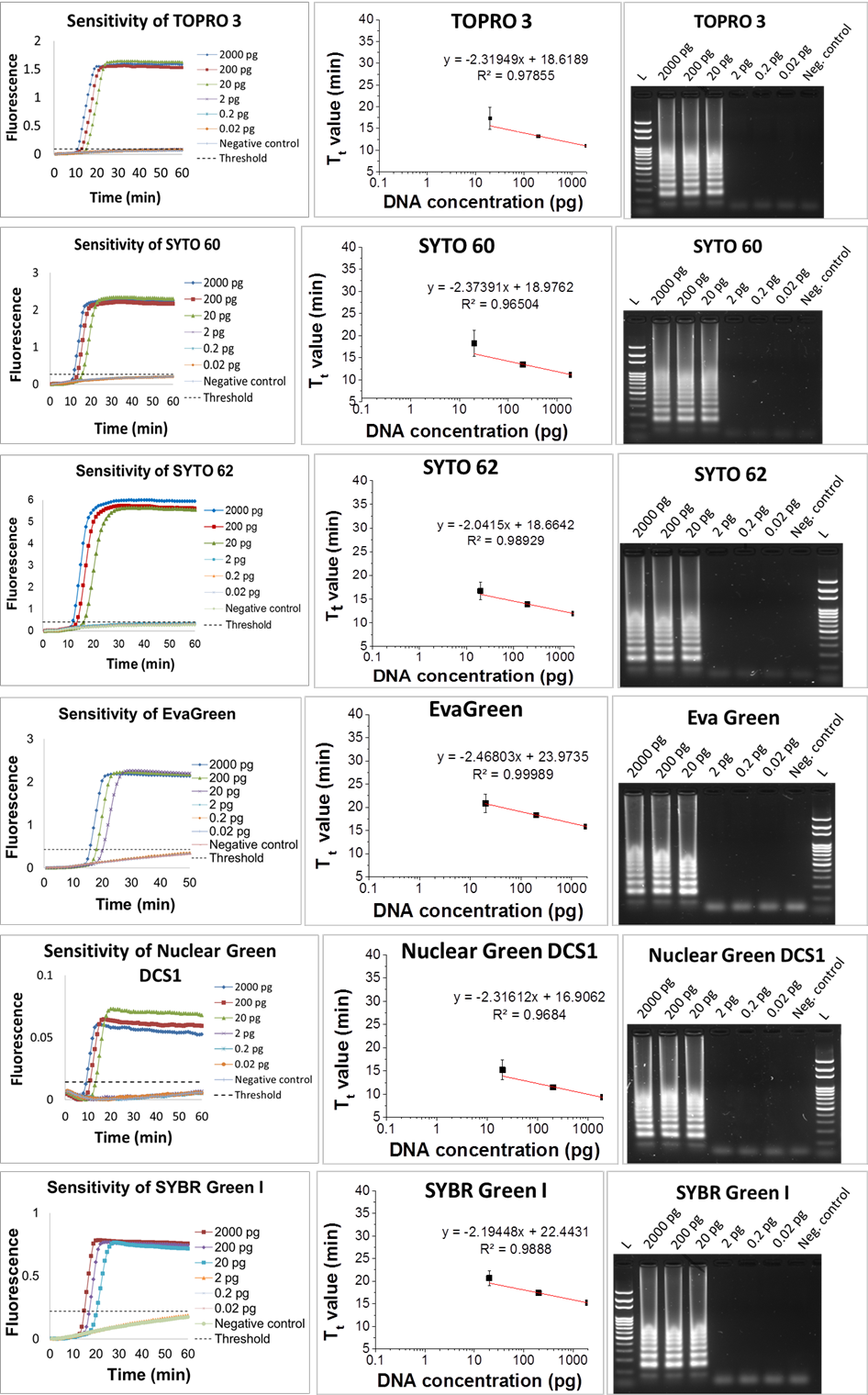


Figure S25. Sensitivity of six dyes in Groups 2 and 3 including TOPRO 3, SYTO 62, SYTO 60, Eva Green, SYBR Green I and Nuclear Green DCS1, which had different degrees of inhibitory effect. The sensitivity test was performed at optimal concentration for each of the dyes. From left to right: Raw fluorescence signal (arbitrary unit), Standard curves and Gel electrophoresis images.


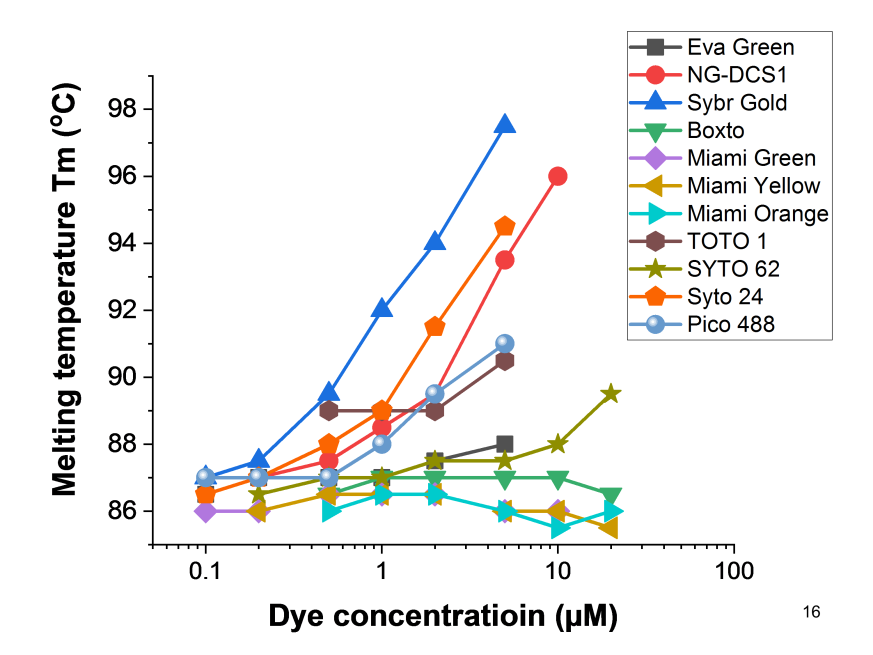


Figure S26. Comparison of dye concentration versus Tm of UC amplicon. The primer sequences and PCR conditions are similar to Haurkur et al. 2007.


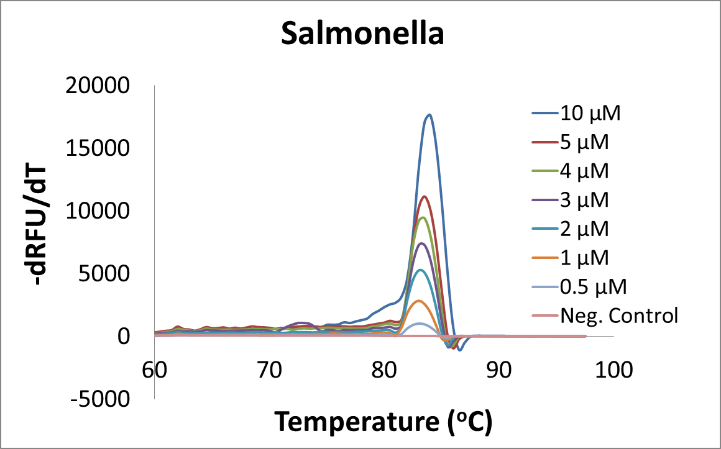

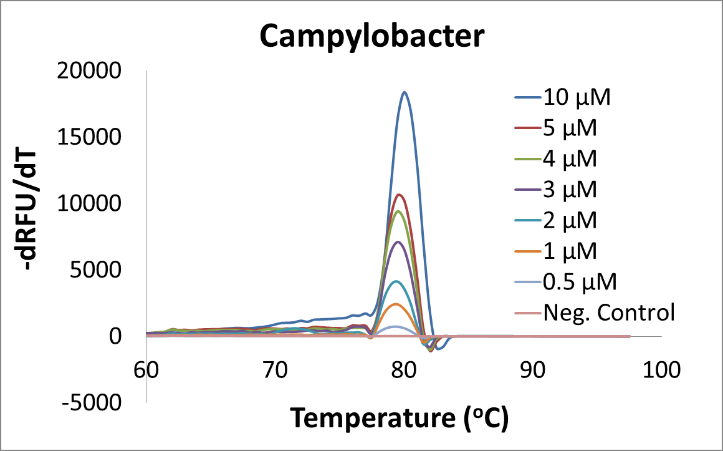


Figure S27. Melting curves of SYTO 9 at different concentrations using *Salmonella* and *Campylobacter* *jejuni* LAMP amplicons. The melting curves were measured in the Piko system after LAMP amplification in FAM channel using 0.5 ^o^C step with a holding time of 10 s at each step from 60-98 ^o^C.


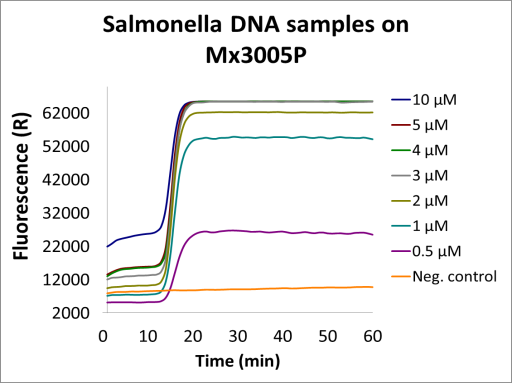

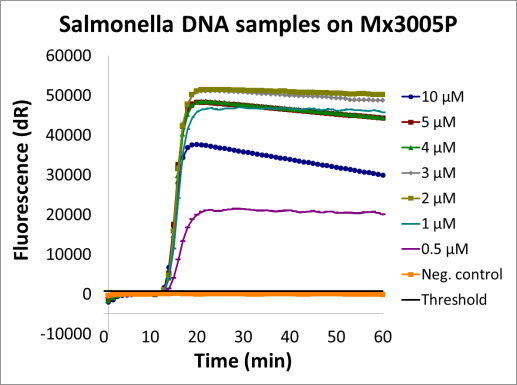

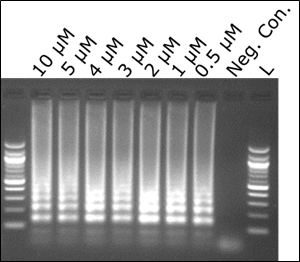


Figure S28. Comparison of fluorescence of *Salmonella* LAMP reaction using DNA samples on the Mx3005P system: A) With background subtraction turned off, B) With background subtraction turned on, and C) Gel electrophoresis image.


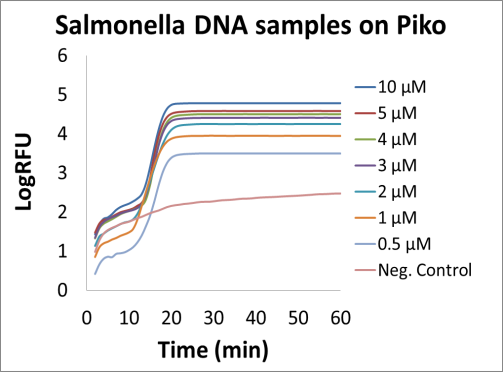

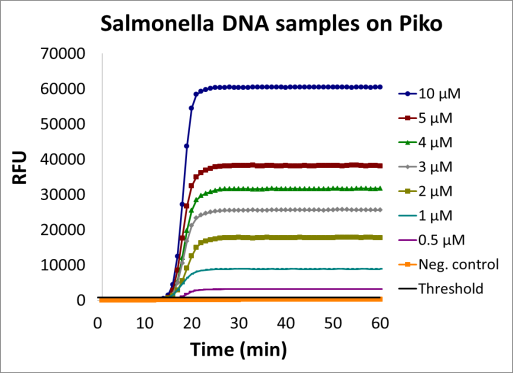

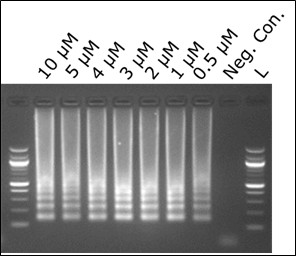


Figure S29. Comparison of fluorescence of *Salmonella* LAMP reaction using DNA samples on the Piko system: A) With background subtraction turned off, B) With background subtraction turned on, and C) Gel electrophoresis image.


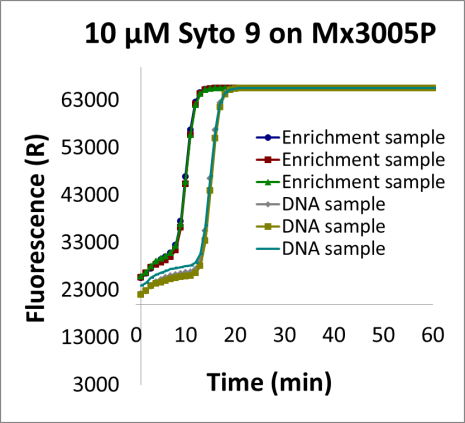

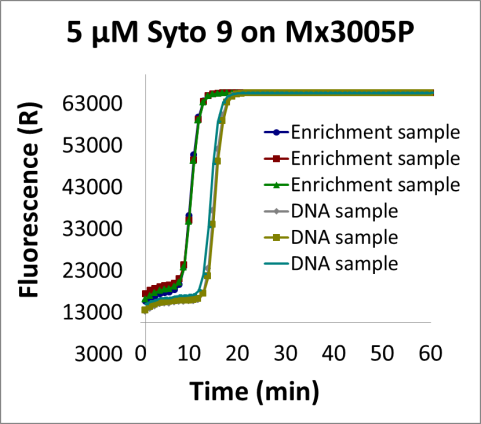

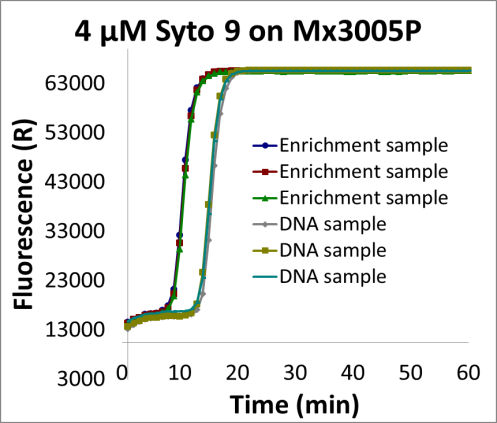


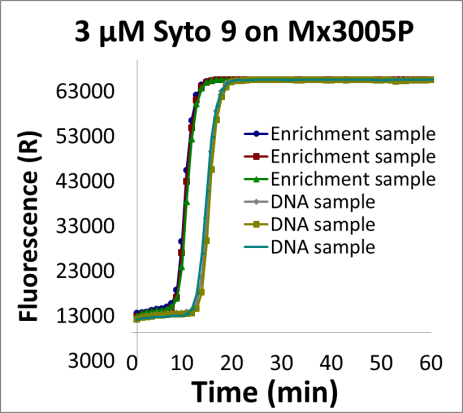

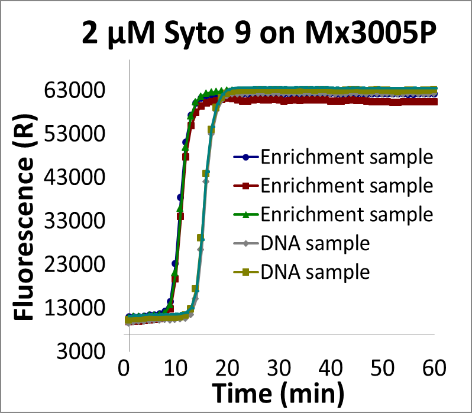

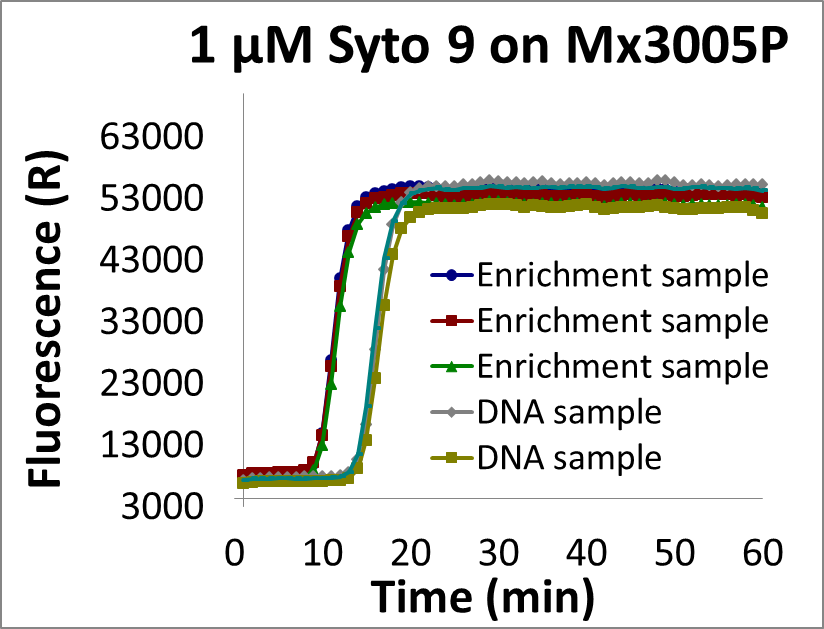


Figure S30. Background fluorescence of samples with different background DNA using varying concentrations of SYTO 9. An enrichment sample was prepared by inoculation of 647 CFU of *Salmonella* Typhimurium in 10 grams of chicken fecal material containing 100 mL of Buffered Peptone Water and incubated at 37 ^o^C for 17 hours. The enrichment sample had a very high concentration of background DNA (approximately 712 ng DNA/uL) due to the growth of the microflora from the sample in the enrichment media.
